# Association Between Fine Particle Waves and Sexual Function: A Nationwide Cross-Sectional Survey in China

**DOI:** 10.3390/toxics13010039

**Published:** 2025-01-06

**Authors:** Weiqian Zhang, Rui Qu, Guan Cheng, Jingxuan Wang, Tailang Yin, Jue Liu, Dongdong Tang, Yan Zhang

**Affiliations:** 1 Reproductive Medicine Center, Renmin Hospital of Wuhan University, Wuhan 430060, China; zhang-wq@whu.edu.cn (W.Z.); qr200111@163.com (R.Q.); w009988776@163.com (J.W.); reproductive@whu.edu.cn (T.Y.); 2 Department of Clinical Laboratory, Renmin Hospital of Wuhan University, Wuhan 430060, China; guanchengst@gmail.com; 3 Department of Epidemiology and Biostatistics, School of Public Health, Peking University, Beijing 100191, China; jueliu@bjmu.edu.cn; 4 Institute for Global Health and Development, Peking University, Beijing 100871, China; 5 Key Laboratory of Epidemiology of Major Diseases, Peking University, Ministry of Education, Beijing 100083, China; 6 NHC Key Laboratory of Study on Abnormal Gametes and Reproductive Tract, Anhui Medical University, Hefei 230032, China; 7 Key Laboratory of Population Health Across Life Cycle, Anhui Medical University, Ministry of Education of the People’s Republic of China, Hefei 230032, China; 8 Reproductive Medicine Center, Department of Obstetrics and Gynecology, the First Affiliated Hospital of Anhui Medical University, Hefei 230022, China

**Keywords:** PM wave, sexual function, depression scores, mediation analysis

## Abstract

Background: The effect of the long-term persistently elevated air pollutants, often referred to as air pollution waves, on sexual function has not been sufficiently addressed. Methods: This nationwide cross-sectional study involved 12,157 participants, with 5496 females and 5039 males. PM waves were characterized by daily average PM concentrations surpassing Grade II thresholds of China’s ambient air quality standards (PM_2.5_ > 75 μg/m^3^, PM_10_ > 150 μg/m^3^) for three or more consecutive days (3–8 days). Male sexual function was assessed through the International Index of Erectile Function-5 (IIEF-5) and the Premature Ejaculation Diagnostic Tool (PEDT), while female sexual function was evaluated using the Female Sexual Function Index (FSFI). A multivariate linear regression model was employed to investigate the link between PM wave exposure and sexual function. Results: Exposure to PM_10_ waves, defined as 3 (β = −0.0145, 95%CI = −0.0280, −0.0010), 4 (β = −0.0145, 95%CI = −0.0280, −0.0010), 5 (β = −0.0193, 95%CI = −0.0371, −0.0015), 6 (β = −0.0218, 95%CI = −0.0415, −0.0021), 7 (β = −0.0243, 95%CI = −0.0458, −0.0028), and 8 (β = −0.0243, 95%CI = −0.0458, −0.0028) consecutive days, negatively impacted IIEF-5 scores and male sexual function. Moreover, depression levels, as evaluated by the PHQ-9, played a mediating role in the connection between PM_10_ waves and IIEF-5 scores. The potentially vulnerable subgroups were the younger 20–30 and the low-income groups. Conclusions: Our results suggest for the first time that PM_10_ waves are associated with decreased IIEF-5 scores, which are mediated by depression score PHQ-9, informing policy formulation for public health interventions and individual safeguarding.

## 1. Introduction

Ambient air pollution represents a significant global public health concern with severe implications for human health [1,2]. Current research predominantly focuses on specific pollutants, investigating both the immediate and delayed effects of their daily exposure levels [3,4,5]. With rapid urbanization and industrialization, protracted severe air pollution episodes are becoming increasingly prevalent. However, the long-term consequences of persistently elevated air pollutants, often referred to as “air pollution waves”, have not been sufficiently addressed. Previous studies have defined air pollution waves as consecutive days with an air pollution index (API) exceeding 100, a condition linked to years of life lost due to non-accidental, cardiovascular, and respiratory deaths [6]. The duration of elevated PM_10_ (PM with an aerodynamic diameter of ≤10 μm) concentrations has been linked to an increased mortality risk in East Asia [7]. Exposure to PM_2.5_ (PM with an aerodynamic diameter of ≤2.5 μm) waves has been connected to boosted risks of unfavorable birth outcomes [8]. Additionally, PM_2.5_ waves may also elevate the risk of hospital admissions for schizophrenia [9]. Investigating the health impacts of sustained high-level air pollution is crucial for developing air pollution control strategies and public health interventions.

Air pollution has been demonstrated to harm human reproductive health. In males, exposure to PM can detrimentally impact sperm motility and semen quality [10,11,12]. Our previous study indicated that exposure to nitrogen dioxide (NO_2_) and PM_2.5_ may negatively influence sexual function among sexually active males in China [13]. For females, PM_2.5_ exposure may lead to ovarian dysfunction and reduced ovarian reserve [14,15]. A nationwide study across China showed that exposure to PM_2.5_ decreased human fecundity, presented by a longer time to pregnancy and higher odds of infertility [16]. However, there are no studies on the relationship between sexual function and PM air pollution waves. Research also has demonstrated a correlation between PM pollution and depression [17], which is a known risk factor for sexual dysfunction [18]. However, the interrelationships among these three variables have not yet been investigated.

In this present study, we conducted a nationwide observational study among the sexually active population in China, aiming to explore the association between PM waves and sexual function. Particular attention was given to the mediating effect of depression in the relationship between ambient PM waves and adverse sexual function.

## 2. Methods

### 2.1. Study Population

**Figure 1:**
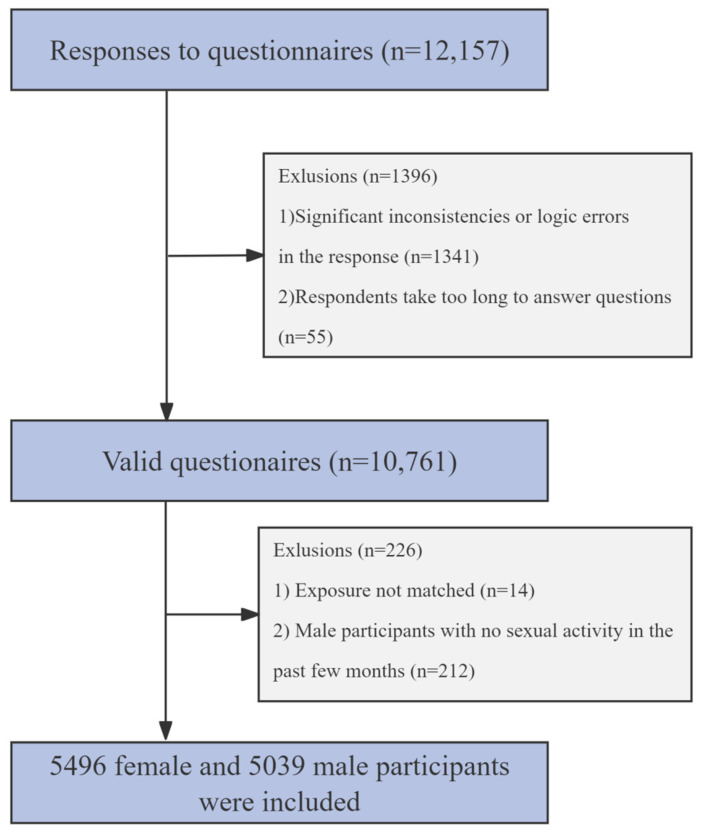
Flowchart of the exclusion criteria for participants in this study.

Utilizing the sex ratios and population compositions of the eastern, central, and western regions as reported in the China Health Statistics Yearbook (https://www.yearbookchina.com, accessed on 24 May 2023), we employed a two-stage quota sampling technique to disseminate an anonymous questionnaire via “Survey Star” (Changsha Ranxing Technology Co., Ltd., Changsha China, https://www.wjx.cn/, accessed on 1 September 2023), a website reaching over 6.2 million individuals. The questionnaire comprised demographic data (age, gender, smoking status, education, income, etc.), and assessments such as the Patient Health Questionnaire-9 (PHQ-9), International Index of Erectile Function-5 (IIEF-5), Premature Ejaculation Diagnostic Tool (PEDT), and Female Sexual Function Index (FSFI) scores. Each account was restricted to a single submission after completing all questions. Between 7 July and 17 August 2023, we collected a total of 12,157 questionnaires, achieving a male-to-female ratio of 1:1, with participant distributions of 5255 in the eastern, 3704 in the central, and 3198 in the western regions, respectively. In the survey’s framework, invitation letters were disseminated across all 31 provinces, elucidating this study’s theme and objectives, and included a link to the online questionnaire. The study’s inclusion criteria were as follows: (1) Being married. (2) Aged between 20 and 40 years. (3) No significant physical or psychological illnesses. (4) No recent medications affecting sexual function or depression-related medications (antidepressants, antipsychotics, anxiolytics, blood pressure medications, hormones, diuretics, antihistamines, etc.). Exclusion criteria are detailed in Figure 1. The study ultimately included 5496 women and 5039 men. The Ethics Committee of the First Affiliated Hospital of Anhui Medical University granted approval for the study.

### 2.2. Assessment of Sexual Function and Depressive State

#### 2.2.1. Sexual Function Assessment Tools

The IIEF-5 and the PEDT scores are recognized as valid metrics for assessing male sexual dysfunction. The IIEF-5, a self-report tool, evaluates erectile dysfunction across five dimensions: erectile function, libido, orgasmic function, intercourse satisfaction, and overall satisfaction. Total scores, derived from summing individual item scores, correlate with greater erectile function and intercourse satisfaction [19]. The PEDT evaluates premature ejaculation in men across five dimensions: ejaculatory control, frequency, minimal sexual stimulation, emotional distress, and interpersonal difficulties. Total scores are calculated by summing the scores for each component, with higher scores indicating an increased likelihood of premature ejaculation [20,21].

The FSFI, a 19-item self-report instrument, is regarded as the gold standard for assessing female sexual functioning [22]. The FSFI evaluates female sexual functioning across six domains: desire, arousal, lubrication, orgasm, satisfaction, and pain, where higher scores denote enhanced sexual functioning.

#### 2.2.2. Depression Assessment Tool

The PHQ-9 is derived from the depression section of the Patient Health Questionnaire (PHQ) developed by Spitzer et al. [23]. It is the most commonly used tool for screening and assessing the severity of depression in general practice [24]. As a severity measure, the PHQ-9 has a score range of 0 to 27, with a standardized threshold score of 10 or more for major depression, with higher scores indicating more severe depressive states [24]. Its validity has been validated in multilingual settings, including Chinese, and across age groups and genders [25,26].

### 2.3. Environmental Information

Based on the China Urban Air Quality Real-Time Dissemination Platform (https://air.cnemc.cn:18007/, accessed on 6 April 2024), we collected day-by-day pollutant data from July 2022–August 2023 at various stations across the country, including PM_10_, PM_2.5_, and NO_2_. Temperatures were obtained through the National Oceanic and Atmospheric Administration’s Global Weather Web site (https://www.ncei.noaa.gov/data/global-summary-of-the-day/archive/, accessed on 8 April 2024). We evaluated individual day-by-day pollutant or temperature exposures using inverse-distance-weighted (IDW) interpolation based on each subject’s residential address in conjunction with the location of the monitoring station. Mean exposure levels were also calculated for 1, 3, 6, and 12 months before exposure.

### 2.4. PM Wave Assessment

To date, there is no clear definition of air pollution waves. Reflecting both intensity and duration, air pollution waves have been defined in some studies as periods of at least two consecutive days where pollutant concentrations surpass absolute or relative thresholds [7,8,9]. Guo et al. characterized the PM_2.5_ wave by the absolute threshold of the secondary limit (75 μg/m^3^) according to the Chinese ambient air quality standard [8]. Building on prior research, this study established 12 definitions for PM_2.5_ and PM_10_ waves, based on exceeding the secondary limits of the Chinese Ambient Air Quality Standards (PM_2.5_ > 75 μg/m^3^; PM_10_ > 150 μg/m^3^) and durations of three or more consecutive days, to assess PM wave exposure in the year preceding the questionnaire completion. These were designated as PM_2.5_-75 μg/m^3^-D3, PM_2.5_-75 μg/m^3^-D4, PM_2.5_-75 μg/m^3^-D5, PM_10_-150 μg/m^3^-D3, PM_10_-150 μg/m^3^-D4, and PM_10_-150 μg/m^3^-D5, respectively.

### 2.5. Covariates

Potential confounding variables were selected using directed acyclic plots (Figure S1) and fine-tuned to the model as confounders in subsequent analyses. Based on the previous literature [27,28,29], the covariates were adjusted as follows: (1) Demographics: age, population, residence, region, and income. (2) Lifestyle: body mass index (BMI), smoking, and alcohol consumption. (3) Health and psychological status: health status, underlying diseases, personality, and depression scores. (4) Environmental factors: NO_2_ concentration in the past 1, 3, and 6 months and average PM_2.5_ concentration in the past 12 months [13].

### 2.6. Statistical Analysis

Baseline characteristics were expressed as n (%) and median (including quartiles). After adjusting for covariates in Section 2.5, multivariate linear regression models were utilized to explore the association between PM_2.5_ and PM_10_ waves and male sexual function (IIEF-5 score and PEDT score) and female sexual function (FSFI score). In addition, we hypothesized that depression score PHQ-9 would mediate the effects of PM wave exposure on sexual function [30]. We used mediation analyses to categorize the overall impact of PM waves on sexual function into direct versus mediated effects and tested the significance of indirect effects by using bootstrapping (1000 simulations). Specifically, we fitted two linear regression models: the first linear regression model estimated the association between PM wave exposure and depression scores. In contrast, the second linear regression model assessed the association between depression scores and sexual functioning scores. The proportion of mediating effects was calculated as (β indirect effect/β total effect) × 100%.

To explore potential influences and identify susceptibility groups, subgroup analyses were conducted by age (<30 years and ≥30 years) and mean monthly income (RMB < 5000, RMB 5000–10,000, and RMB > 10,000). Sensitivity analyses were performed by adjusting for the mean temperature 1 year before completion of the questionnaire.

All analyses were performed using R4.3.2 (Rstudio Inc., New Haven, CT, USA). Statistical tests were two-sided, and a *p* value of less than 0.05 was considered statistically significant.

## 3. Results

### 3.1. Characteristics of the Study Population

The demographic characteristics of the study participants are shown in Table 1. Among the 5496 female participants, the median age was 28 years (IQR: 25.00–32.00), the median depression score was 6 (IQR: 4.00–10.00), and the median the Female Sexual Function Index (FSFI) score was 24.5 (IQR: 19.90–28.10). Higher proportions of Han Chinese (94.2%), non-smokers (88.8%), non-drinkers (68.3%), and bachelor’s degree (84.3%) were found. Among the 5039 male participants, the median age was 29 years (IQR: 25.00–33.00), the median depression score was 6 (IQR: 4.00–9.00), the median the International Index of Erectile Function-5 (IIEF-5) score was 21.00 (IQR: 18.00–23.00), and the median Premature Ejaculation Diagnostic Tool (PEDT) score was 10 (IQR: 8.00–13.00). Higher proportions of Han Chinese (94.4%), normal BMI (67.5%), no underlying disease (77.8%), and urban areas (92.0%) were observed.

### 3.2. Overall Association

**Figure 2:**
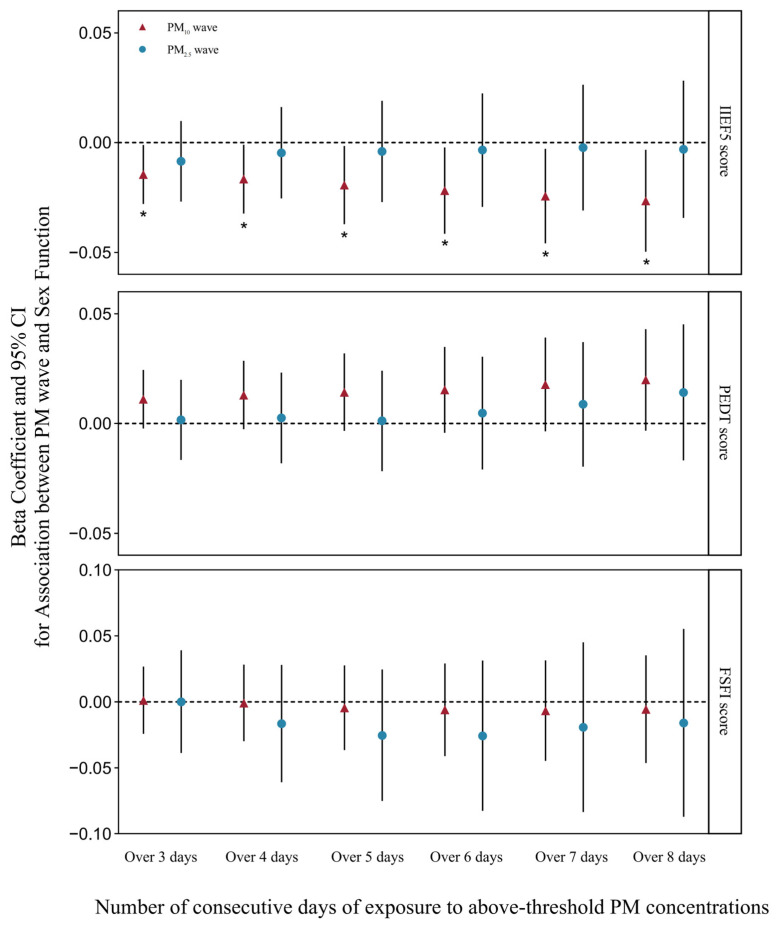
Association of sexual function scores with PM_2.5_ and PM_10_ waves under different definitions. The models were adjusted for age, population, residence, region, income, BMI, smoking status, drinking status, personality, constitution, underlying disease, depression score, mean NO_2_ exposure in the first 1, 3, and 6 months, and mean PM_2.5_ exposure in the first 12 months. PM = particulate matter. * *p*  <  0.05.

After controlling for variables such as age, body mass index, depression score, smoking and drinking habits, education, income, residency, region, personality, physical fitness, underlying diseases, NO_2_ exposure levels in the past 1, 3, and 6 months, and PM_2.5_ exposure levels in the past 12 months, we employed multiple linear regression models to evaluate the relationship between PM_2.5_ and PM_10_ waves over the previous year and sexual functioning in both males and females (see Table S1). Results indicated that exposure to PM_10_ waves, defined as 3 to 8 consecutive days, negatively impacted IIEF-5 scores and male sexual function (β = −0.0145, 95%CI = −0.0280,−0.0010; β = −0.0166, 95%CI = −0.0323, −0.0009; β = −0.0193, 95%CI = −0.0371, −0.0015; β = −0.0218, 95%CI = −0.0415, −0.0021; β = −0.0243, 95%CI = −0.0458, −0.0028; β = −0.0264, 95%CI = −0.0497, −0.0032). PM_10_ waves did not significantly affect PEDT and FSFI scores. PM_2.5_ waves were not found to significantly impair sexual function in either males or females (Figure 2).

### 3.3. Mediation Analysis

**Figure 3:**
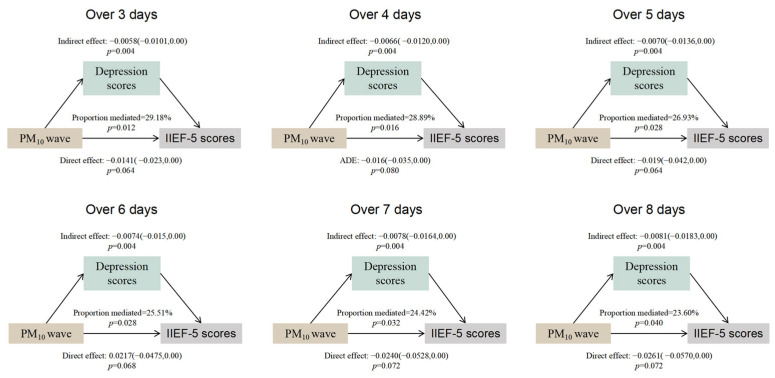
Mediation analysis of depression scores in the association between PM_10_ waves and IIEF-5 scores. The models were adjusted for age, population, residence, region, income, BMI, smoking status, drinking status, personality, constitution, underlying disease, depression score, mean NO_2_ exposure in the first 1, 3, and 6 months, and mean PM_2.5_ exposure in the first 12 months. PM = particulate matter.

Given that mediation analysis requires a statistically significant total effect, our investigation was limited to the mediating role of the PHQ-9 score between PM10 wave exposure and IIEF-5 scores. We found that PHQ-9 significantly mediated the effects of PM_10_-150 μg/m^3^-D3 (mediated proportion = 29.18%, *p* = 0.012), PM_10_-150 μg/m^3^-D4 (mediated proportion = 28.89%, *p* = 0.016), PM_10_-150 μg/m^3^-D5 (mediated proportion = 26.93%, *p* = 0.028), PM_10_-150 μg/m^3^-D6 (mediated proportion = 25.51%, *p* = 0.028), PM_10_-150 μg/m^3^-D7 (mediated proportion = 24.42%, *p* = 0.032), and PM_10_-150 μg/m^3^-D8 (mediated proportion = 23.60%, *p* = 0.040) under the definition of PM_10_ waves and IIEF-5 score (Figure 3).

### 3.4. Subgroup and Sensitivity Analysis

**Figure 4:**
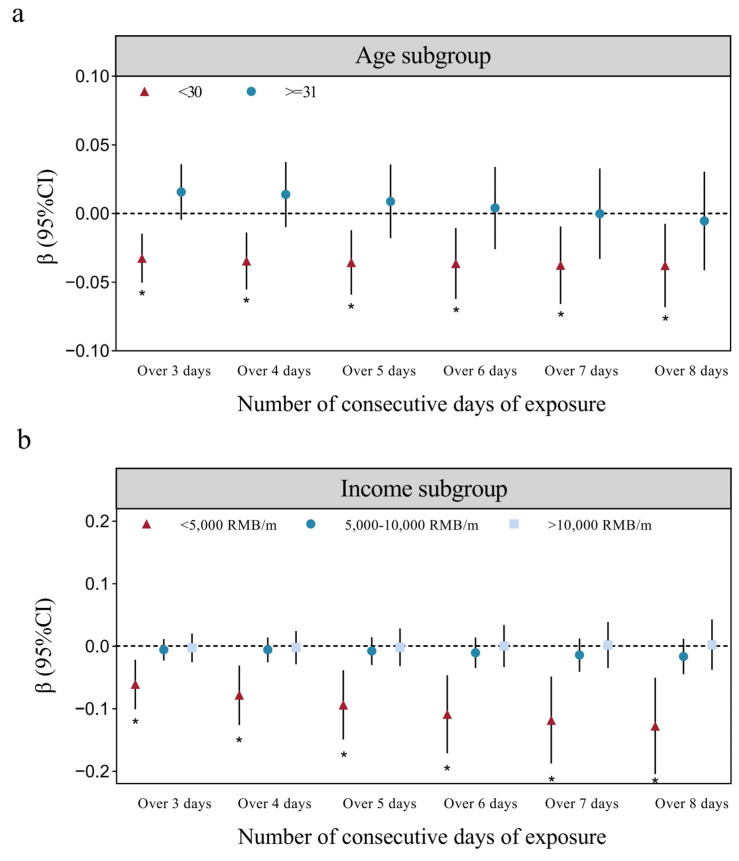
Effect of PM_10_ wave exposure on IIEF-5 scores in different subgroups. (**a**) Age subgroup. (**b**) Income subgroup. The models were adjusted for age, population, residence, region, income, BMI, smoking status, drinking status, personality, constitution, underlying disease, depression score, mean NO_2_ exposure in the first 1, 3, and 6 months, and mean PM_2.5_ exposure in the first 12 months. * *p*  <  0.05.

The results of the subgroup analysis are presented in Tables S2 and S3. IIEF-5 scores in male participants aged 20–30 were more susceptible to PM10 wave exposure compared to those aged 31–40 (Figure 4a). Furthermore, the association between IIEF-5 scores and PM_10_ wave exposure was stronger among male participants with lower income (<5000 RMB/month) (Figure 4b). The inclusion of the average temperature over the previous 12 months as a covariate in the sensitivity analyses did not significantly alter our results (Table 2).

## 4. Discussion

Our results demonstrated for the first time that PM_10_ waves defined as 3–8 consecutive days were associated with decreased IIEF-5 scores after adjusting for covariates, which was mediated by depression score PHQ-9. Moreover, the potentially vulnerable subgroups were the younger 20–30 and the low-income group.

To date, only a limited number of studies have investigated the long-term effects of prolonged exposure to elevated levels of ambient air pollutants. They focused on mortality, years of life lost, hospital admissions for schizophrenia, and adverse birth outcomes [6,7,8,9]. In contrast to the previous research, we investigated the durational impact of PM_2.5_ and PM_10_ waves on sexual function through the mediation of depression scores. Research has varied in its definition of ambient air pollution waves. Some adopted the threshold quantities of ambient air pollutants, such as PM_10_ > 75 μg/m^3^ or API >100 [6,7]. Some countered that different places have varied levels of ambient air pollution and population acclimatization; therefore, picking a single concentration as the threshold is incorrect [9]. In this study, we defined PM waves as a daily average PM concentration exceeding the Grade II limits of China’s ambient air quality standards (PM_2.5_ > 75 μg/m^3^, PM_10_ > 150 μg/m^3^) for at least three or more consecutive days (3, 4, 5, 6, 7, and 8 days). Adopting Chinese air quality standards and defining them with thresholds makes the results more informative and uniform. In addition, the estimated impacts of more varied PM_2.5_ and PM_10_ waves can be observed in this study.

In this study, we first conducted the sexual function assessment using quantitative tools (PEDT, IIEF-5, and FSFI scores) in a nationwide sample of sexually active individuals aged 20–40 years. The results are roughly in line with previous studies. A cross-sectional survey conducted across five regions of China, involving 1239 men aged 18–60 years, demonstrated that the median scores for the IIEF-5 and PEDT were 20.00 (IQR: 13.00–22.00) and 9.00 (IQR: 5.50–13.00), respectively [31]. Additionally, an epidemiological study in Beijing, China, which included 4697 sexually active women aged 20–60 years, reported a mean FSFI score of 23.92 ± 6.37 [32]. Although results of PEDT, IIEF-5, and FSFI scores in the Chinese population may have nuance across studies due to distinctions in inclusion criteria, the PEDT, IIEF-5, and FSFI scores continue to be a valid tool to quantify sexual functioning [33,34]. Further large-sample nationwide investigation is necessary to determine the status of PEDT, IIEF-5, and FSFI scores in the general population across China.

Recent investigations have suggested that air pollution may contribute to the pathogenesis of erectile dysfunction. A former study indicated that exposure to PM_2.5_, NO_2_, and O_3_ would affect the incidence of erectile dysfunction among participants aged 57–85, but did not reach nominal statistical significance [35]. Our preceding result documented declined IIEF-5 scores under NO_2_ exposure among Chinese sexually active males [13]. We demonstrated for the first time that sustained high-level PM_10_ (PM_10_ waves) was associated with decreased IIEF-5 scores. Though the underlying mechanisms remain elusive at present, it has been proposed that air pollution may interfere with the hypothalamic–pituitary–testicular axis and decrease the synthesis of testosterone [36,37,38], thereby reducing erectile function [39,40]. In addition, the vascular endothelium is deemed a pivotal factor in the pathogenesis of erectile dysfunction [41,42]. The downregulation of endothelial nitric oxide synthase (NOS) expression and activity within the penile cavernous tissue by pollutant exposure may represent a significant risk factor for erectile dysfunction [43,44]. The implicated mechanism of the above effects may include excessive production of reactive oxygen species and initiation of inflammation, decreased antioxidant bioavailability, and development of oxidative stress [45,46].

Erectile dysfunction also encompasses psychogenic components. Existing evidence has verified the bidirectional relationship between depression and erectile dysfunction [18]. Large cohort studies and systematic reviews of global evidence have also demonstrated the relationship between mental health disorders and exposure to long- or short-term air pollution [47,48]. Increased incident risk and daily hospital admission for depression were associated with short-term exposure to PM_10_ [49,50]. However, there is a dearth of studies elucidating the interplay among air pollution, depression, and erectile dysfunction, particularly regarding the potential mediating influence of depression. Our results found that depression score PHQ-9 significantly mediated the association between PM_10_ waves and IIEF-5 scores under the 3–8 day definition, offering potential etiologic information for the impairment of erectile function by PM_10_ waves. The central nervous system is particularly vulnerable to PM due to its elevated metabolic demands, limited endogenous antioxidant capacities, and heightened energy consumption. Exposure to pollutants can induce stress hormone-dependent signaling pathways. PM stimulates the hypothalamic–pituitary–adrenal (HPA) axis, and increased glucocorticoid levels have been linked to adverse neurobehavioral effects [51]. PM also exacerbates depression via inflammatory and oxidative stress. Elevated oxidative stress within the nervous system proves detrimental to dopaminergic neurons, leading to the onset of depression [50]. PM serves as an inflammatory trigger, stimulating cytokines and inflammatory signaling molecules, thereby precipitating depression and associated conditions such as erectile dysfunction [52,53]. The relationship between mental health and sexual dysfunction is bidirectional and complex. Sexual dysfunction can precipitate psychological issues in individuals, while pre-existing psychological problems may intensify sexual dysfunction. This dynamic interaction often results in a pernicious cycle. More animal experiments and population investigations are imperative to elucidate the potential underlying mechanisms.

In the subgroup analysis, we found the potentially vulnerable subgroups for PM_10_ wave exposure regarding male sexual function were the younger group aged 20–30 and the low-income group. Prior studies have revealed that aging is a risk factor, with a dramatic increase in the prevalence of erectile dysfunction after the age of 40 [54]. The imbalance in the proportion of participants in different age groups, 61.1% of subjects were aged 20–30 while only 38.9% were aged 31–40, may have resulted in our outcome. Previous studies have proved that low socioeconomic status measuring with the poverty/income ratio may be associated with erectile dysfunction, consistent with our results [55,56]. Low-income populations have been exposed to higher average PM_2.5_ levels than high-income ones [57,58], possibly due to longer outdoor working hours and polluting solid fuels for cooking and heating [59]. Low income was also a sustained stress source and the cumulative poor physical function and emotional depression from chronic stress is related to erectile dysfunction.

Compared with unchangeable pathogenic factors such as gene defects [60,61,62], sexual dysfunction induced by air pollution exposure could be prevented and reversed via policy-based interventions and individual protections. Governments could enact early warning systems for air pollution and facilitate energy-saving and emission reduction measures. Low-income groups need focused attention. Governments need to strengthen policy support for low-income people to replace solid polluting fuels with cleaner ones as soon as possible. Moreover, individuals could employ strategies such as utilizing air purifiers or minimizing outdoor activities during periods of heightened PM_10_ levels. Our study bears significant implications for public health.

This is the first study to assess the relationship between sexual function and prolonged exposure to high particulate matter over an extended period of days. The strength of our study resides in the incorporation of a nationwide representative population of sexually active individuals and the employment of quantitative tools, such as PEDT, IIEF-5, and FSFI scores, for sexual function evaluation. Furthermore, the exclusion of subjects recently taking medications affecting sexual function or depression-related medications (antidepressants, antipsychotics, anxiolytics, blood pressure medications, etc.) eliminated confounding from these drugs, increasing the credibility of this study. Nonetheless, there are several limitations. Firstly, occupation and lifestyle were not adequately considered, potentially leading to inaccurate assessments of air pollution exposure. Moreover, our population primarily consisted of Asian Chinese ethnicity, which may restrict the generalizability of our findings to other demographic groups. In addition, the questionnaire lacked assessment of hormone imbalances. The online survey may be filled out by more patients interested in sexual dysfunction than the general population, which may lead to bias.

## 5. Conclusions

Our results suggest for the first time that PM_10_ waves are associated with decreased IIEF-5 scores, which are mediated by depression score PHQ-9. The potentially vulnerable subgroups were those aged 20–30 and those with low income. Our findings offer insights into preventing or reversing sexual dysfunction amid sustained high PM_10_ air pollution concentrations, informing policy formulation for public health interventions and individual safeguarding.

## Figures and Tables

**Table 1:** Characteristics of the study population.

Characteristic	Female (n = 5496)	Male (n = 5039)
age (median [IQR])	28 (25, 32)	29 (25, 33)
population (%)		
Han Chinese	5176 (94.1776)	4758 (94.4235)
Non-Han Chinese	320 (5.8224)	281 (5.5765)
BMI_grade (%)		
<18.5	1385 (25.2001)	390 (7.7396)
18.5–24.9	3624 (65.9389)	3399 (67.4539)
>24.9	487 (8.8610)	1250 (24.8065)
drinking (%)		
Never	3756 (68.3406)	1568 (31.1173)
Former	330 (6.0044)	523 (10.3790)
Current	1410 (25.6550)	2948 (58.5037)
smoking (%)		
Never	4883 (88.8464)	2326 (46.1600)
Former	249 (4.5306)	724 (14.3679)
Current	364 (6.6230)	1989 (39.4721)
constitution (%)		
Poor	343 (6.2409)	250 (4.9613)
Moderate	3464 (63.0277)	2984 (59.2181)
Good	1689 (30.7314)	1805 (35.8206)
underlying_disease (%)		
Absence	4484 (81.5866)	3919 (77.7734)
Presence	1012 (18.4134)	1120 (22.2266)
education (%)		
Secondary education or lower	333 (6.0590)	447 (8.8708)
Undergraduate level	4634 (84.3158)	4025 (79.8770)
Postgraduate level or higher	529 (9.6252)	567 (11.2522)
income (%)		
<5000/m	1736 (31.5866)	1012 (20.0833)
5000–10,000/m	2853 (51.9105)	2751 (54.5942)
>10,000/m	907 (16.5029)	1276 (25.3225)
resident (%)		
Urban areas	5000 (90.9753)	4635 (91.9825)
Rural areas	496 (9.0247)	404 (8.0175)
region (%)		
Eastern Region	2465 (44.8508)	2123 (42.1314)
Central Region	1680 (30.5677)	1499 (29.7480)
Western Region	1351 (24.5815)	1417 (28.1206)
personality (%)		
Introverted	2812 (51.1645)	2359 (46.8148)
Extroverted	2684 (48.8355)	2680 (53.1852)
depression_score (median [IQR])	6 (4, 10)	6 (4, 9)
FSFI_score (median [IQR])	24.5000 (19.9000, 28.1000)	
IIEF-5_score (median [IQR])		21 (18, 23)
PEDT_score (median [IQR])		10 (8, 13)

Note: IQR: interquartile range; BMI: body mass index.

**Table 2:** Sensitivity analysis of the association of sexual function scores with PM_2.5_ and PM_10_ waves under different definitions.

PM Wave	Male				Female	
	IIEF-5 Scores		PEDT Scores		FSFI Scores	
	β (95%CI)	*p*	β (95%CI)	*p*	β (95%CI)	*p*
PM_2.5_-75 μg/m^3^-D3	−0.0084 (−0.0270, 0.0102)	0.3769	0.0019 (−0.0166, 0.0203)	0.8420	0.0077 (−0.0316, 0.0470)	0.7008
PM_2.5_-75 μg/m^3^-D4	−0.0044 (−0.0257, 0.0169)	0.6867	0.0030 (−0.0182, 0.0241)	0.7840	−0.0038 (−0.0493, 0.0417)	0.8699
PM_2.5_-75 μg/m^3^-D5	−0.0036 (−0.0274, 0.0201)	0.7636	0.0017 (−0.0219, 0.0252)	0.8897	−0.0096 (−0.0611, 0.0418)	0.7131
PM_2.5_-75 μg/m^3^-D6	−0.0030 (−0.0296, 0.0236)	0.8260	0.0054 (−0.0210, 0.0318)	0.6878	−0.0084 (−0.0669, 0.0501)	0.7780
PM_2.5_-75 μg/m^3^-D7	−0.0018 (−0.0309, 0.0274)	0.9051	0.0095 (−0.0195, 0.0384)	0.5213	−0.0017 (−0.0673, 0.0640)	0.9598
PM_2.5_-75 μg/m^3^-D8	−0.0026 (−0.0343, 0.0290)	0.8720	0.0149 (−0.0165, 0.0463)	0.3531	0.0002 (−0.0721, 0.0725)	0.9950
PM_10_-150 μg/m^3^-D3	−0.0149 (−0.0284, −0.0013)	0.0316 *	0.0112 (−0.0023, 0.0246)	0.1042	−0.0031 (−0.0288, 0.0225)	0.8102
PM_10_-150 μg/m^3^-D4	−0.0167 (−0.0324, −0.0010)	0.0375 *	0.0130 (−0.0026, 0.0286)	0.1032	−0.0034 (−0.0324, 0.0257)	0.8206
PM_10_-150 μg/m^3^-D5	−0.0193 (−0.0371, −0.0015)	0.0333 *	0.0143 (−0.0034, 0.0319)	0.1125	−0.0058 (−0.0379, 0.0263)	0.7223
PM_10_-150 μg/m^3^-D6	−0.0218 (−0.0414, −0.0021)	0.0302 *	0.0154 (−0.0041, 0.0349)	0.1222	−0.0063 (−0.0413, 0.0286)	0.7229
PM_10_-150 μg/m^3^-D7	−0.0242 (−0.0458, −0.0027)	0.0275 *	0.0179 (−0.0035, 0.0393)	0.1011	−0.0062 (−0.0442, 0.0318)	0.7477
PM_10_-150 μg/m^3^-D8	−0.0264 (−0.0497, −0.0031)	0.0263 *	0.0200 (−0.0031, 0.0431)	0.0898	−0.0053 (−0.0461, 0.0355)	0.7996

Note: the models were adjusted for age, population, residence, region, income, BMI, smoking status, drinking status, personality, constitution, underlying disease, depression score, mean NO_2_ exposure in the first 1, 3, and 6 months, mean PM_2.5_ exposure in the first 12 months, and mean temperature in the first 12 months. * *p*  <  0.05.

## Data Availability

The raw data supporting the conclusions of this article will be made available by the authors on request.

## Supplementary Materials

The following supporting information can be downloaded at: https://www.mdpi.com/article/10.3390/toxics13010039/s1, Figure S1: Directed Acyclic Graph of this study; Table S1: Association of sexual function scores with PM_2.5_ and PM_10_ waves under different definitions; Table S2: Effect of PM_10_ wave exposure on IIEF-5 scores in different age subgroups; Table S3: Effect of PM_10_ wave exposure on IIEF-5 scores in different income subgroups.
