# Supplementary material for: Association Between Fine Particle Waves and Sexual Function: A Nationwide Cross-Sectional Survey in China
Source: Toxics. 2025 Jan 6;13(1):39. doi: 10.3390/toxics13010039 (PMC11769323; doi:10.3390/toxics13010039)
Supplement: Supplementary file 1 [file toxics-13-00039-s001.zip › toxics-3360858-supplementary.pdf]

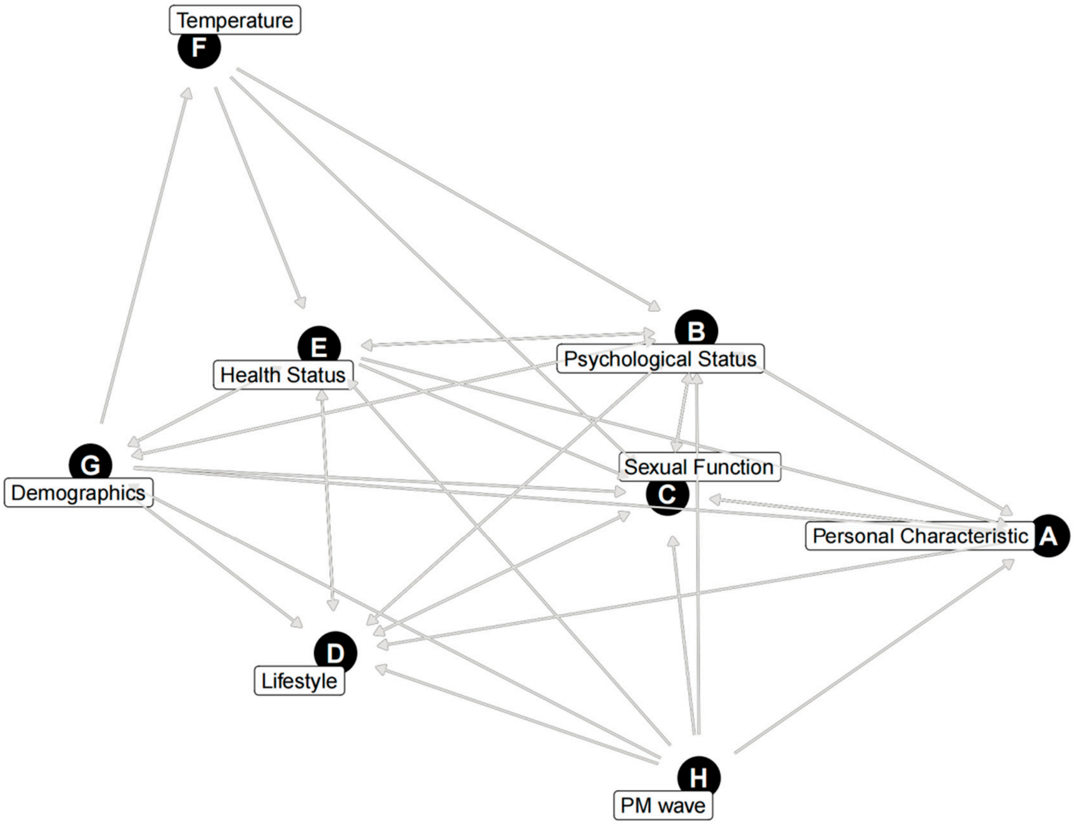

Supplementary Figure S1: Directed Acyclic Graph of this study. This graph represents the hypothesized relationships among various factors related to sexual function. Each node in the graph represents a specific variable: “Demographics (Age, Population, Residence, Region, and Income)”, “Lifestyle (BMI, smoking status, and drinking status)”, “Personal Characteristics (Personality)”, “Health Status (Constitution and underlying disease)”, “Psychological Status (Depression score)”, “Temperature”, “PM wave”, and “Sexual Function”.

Supplement Table S1: Association of sexual function scores with $PM_{2.5}$ and PM_{10} waves under different definitions

	Male				Female	
	IIEF5 scores		PEDT scores		FSFI scores	
PM wave	β(95%CI)	P	β(95%CI)	P	β(95%CI)	P
$PM_{2.5}$-75μg/m^3-D3	-0.0085(-0.0268,0.0099)	0.3675	0.0017(-0.0166,0.0199)	0.8580	0.0002(-0.0388,0.0391)	0.9937
$PM_{2.5}$-75μg/m^3-D4	-0.0046(-0.0254,0.0162)	0.6644	0.0025(-0.0181,0.0232)	0.8097	-0.0165(-0.0609,0.0279)	0.4669
$PM_{2.5}$-75μg/m^3-D5	-0.0040(-0.0270,0.0191)	0.7367	0.0012(-0.0217,0.0241)	0.9163	-0.0254(-0.0752,0.0245)	0.3182
$PM_{2.5}$-75μg/m^3-D6	-0.0034(-0.0293,0.0225)	0.7981	0.0048(-0.0209,0.0305)	0.7162	-0.0257(-0.0826,0.0312)	0.3756
$PM_{2.5}$-75μg/m^3-D7	-0.0022(-0.0308,0.0264)	0.8785	0.0088(-0.0196,0.0372)	0.5451	-0.0192(-0.0834,0.0451)	0.5592
$PM_{2.5}$-75μg/m^3-D8	-0.0030(-0.0342,0.0282)	0.8503	0.0142(-0.0168,0.0452)	0.3699	-0.0159(-0.0872,0.0553)	0.6614
PM_{10}-150μg/m^3-D3	-0.0145(-0.0280,-0.0010)	0.0347*	0.0111(-0.0023,0.0244)	0.1035	0.0013(-0.0242,0.0267)	0.9228
PM_{10}-150μg/m^3-D4	-0.0166(-0.0323,-0.0009)	0.0386*	0.0130(-0.0026,0.0286)	0.1022	-0.0007(-0.0297,0.0282)	0.9598
PM_{10}-150μg/m^3-D5	-0.0193(-0.0371,-0.0015)	0.0334*	0.0143(-0.0033,0.0319)	0.1123	-0.0045(-0.0366,0.0276)	0.7834
PM_{10}-150μg/m^3-D6	-0.0218(-0.0415,-0.0021)	0.0299*	0.0154(-0.0042,0.0349)	0.1229	-0.0060(-0.0410,0.0290)	0.7365
PM_{10}-150μg/m^3-D7	-0.0243(-0.0458,-0.0028)	0.0271*	0.0178(-0.0036,0.0392)	0.1024	-0.0067(-0.0447,0.0314)	0.7309
PM_{10}-150μg/m^3-D8	-0.0264(-0.0497,-0.0032)	0.0259*	0.0199(-0.0032,0.0430)	0.0909	-0.0055(-0.0463,0.0353)	0.7912

Note: The models were adjusted for age, population, residence, region, income, BMI, smoking status, drinking status, personality, constitution, underlying disease,

depression score, mean NO_2 exposure in the first 1, 3, and 6 months, and mean $PM_{2.5}$ exposure in the first 12 months. *$P < 0.05$.

Supplement Table S2. Effect of PM_{10} wave exposure on IIEF-5 scores in different age subgroups

	age≤30 years		age>30 years	
PM_{10} wave	β(95%CI)	P	β(95%CI)	P
PM_{10}-150μg/m^3-D3	-0.0325(-0.0503,-0.0147)	0.0004*	0.0158(-0.0044,0.0360)	0.1249
PM_{10}-150μg/m^3-D4	-0.0345(-0.0553,-0.0138)	0.0011*	0.0139(-0.0098,0.0376)	0.2502
PM_{10}-150μg/m^3-D5	-0.0356(-0.0591,-0.0122)	0.0029*	0.0089(-0.0180,0.0358)	0.5159
PM_{10}-150μg/m^3-D6	-0.0364(-0.0622,-0.0105)	0.0059*	0.0040(-0.0259,0.0340)	0.7930
PM_{10}-150μg/m^3-D7	-0.0376(-0.0658,-0.0093)	0.0091*	-0.0001(-0.0330,0.0329)	0.9973
PM_{10}-150μg/m^3-D8	-0.0379(-0.0683,-0.0074)	0.0148*	-0.0054(-0.0412,0.0304)	0.7691

Note: The models were adjusted for age, population, residence, region, income, BMI, smoking status, drinking status, personality, constitution, underlying disease, depression score, mean NO_2 exposure in the first 1, 3, and 6 months, and mean $PM_{2.5}$ exposure in the first 12 months. PM=particulate matter. *$P < 0.05$.

Supplement Table S3. Effect of PM_{10} wave exposure on IIEF-5 scores in different income subgroups

	Income level					
	<5,000 RMB/m		5,000-10,000 RMB/m		10,000 RMB/m	
PM_{10} wave	β(95%CI)	P	β(95%CI)	P	β(95%CI)	P
PM_{10}-150μg/m^3-D3	-0.0613(-0.1008,-0.0218)	0.0024*	-0.0058(-0.0229,0.0114)	0.5092	-0.0028(-0.0258,0.0202)	0.8118
PM_{10}-150μg/m^3-D4	-0.0785(-0.1260,-0.0310)	0.0012*	-0.0059(-0.0258,0.0140)	0.5625	-0.0024(-0.0289,0.0241)	0.8583
PM_{10}-150μg/m^3-D5	-0.0940(-0.1492,-0.0388)	0.0009*	-0.0080(-0.0303,0.0143)	0.4830	-0.0020(-0.0321,0.0281)	0.8960
PM_{10}-150μg/m^3-D6	-0.1088(-0.1711,-0.0465)	0.0006*	-0.0106(-0.0351,0.0139)	0.3947	0.0002(-0.0333,0.0338)	0.9886
PM_{10}-150μg/m^3-D7	-0.1182(-0.1878,-0.0486)	0.0009*	-0.0143(-0.0409,0.0123)	0.2916	0.0019(-0.0350,0.0388)	0.9199
PM_{10}-150μg/m^3-D8	-0.1277(-0.2045,-0.0508)	0.0012*	-0.0167(-0.0452,0.0118)	0.2508	0.0022(-0.0380,0.0425)	0.9136

Note: The models were adjusted for age, population, residence, region, income, BMI, smoking status, drinking status, personality, constitution, underlying disease, depression score, mean NO_2 exposure in the first 1, 3, and 6 months, and mean $PM_{2.5}$ exposure in the first 12 months. PM=particulate matter. *$P < 0.05$.
